# EPMA position paper in cancer: current overview and future perspectives

**DOI:** 10.1186/s13167-015-0030-6

**Published:** 2015-04-15

**Authors:** Godfrey Grech, Xianquan Zhan, Byong Chul Yoo, Rostyslav Bubnov, Suzanne Hagan, Romano Danesi, Giorgio Vittadini, Dominic M Desiderio

**Affiliations:** Department of Pathology, Faculty of Medicine and Surgery, University of Malta, Msida, Malta; Key Laboratory of Cancer Proteomics of Chinese Ministry of Health, Xiangya Hospital, Central South University, Changsha, China; Colorectal Cancer Branch, Division of Translational and Clinical Research I, Research Institute, National Cancer Center, Gyeonggi, 410-769 Republic of Korea; Clinical Hospital ‘Pheophania’ of State Management of Affairs Department, Kyiv, Ukraine; Zabolotny Institute of Microbiology and Virology, National Academy of Sciences of Ukraine, Kyiv, Ukraine; Dept of Life Sciences, School of Health and Life Sciences, Glasgow Caledonian University, Glasgow, UK; Department of Clinical and Experimental Medicine, University of Pisa, Pisa, Italy; Bracco Imaging, Centro Ricerche Bracco, San Donato Milanese, Italy; Department of Neurology, University of Tennessee Center for Health Science, Memphis, USA

**Keywords:** Predictive preventive personalized medicine, Risk assessment, Expert recommendation, Standardization, Individual profile, Disease modeling, Multimodal diagnostics, Screening, Biomarker, Biobank

## Abstract

At present, a radical shift in cancer treatment is occurring in terms of predictive, preventive, and personalized medicine (PPPM). Individual patients will participate in more aspects of their healthcare. During the development of PPPM, many rapid, specific, and sensitive new methods for earlier detection of cancer will result in more efficient management of the patient and hence a better quality of life. Coordination of the various activities among different healthcare professionals in primary, secondary, and tertiary care requires well-defined competencies, implementation of training and educational programs, sharing of data, and harmonized guidelines. In this position paper, the current knowledge to understand cancer predisposition and risk factors, the cellular biology of cancer, predictive markers and treatment outcome, the improvement in technologies in screening and diagnosis, and provision of better drug development solutions are discussed in the context of a better implementation of personalized medicine. Recognition of the major risk factors for cancer initiation is the key for preventive strategies (EPMA J. 4(1):6, 2013). Of interest, cancer predisposing syndromes in particular the monogenic subtypes that lead to cancer progression are well defined and one should focus on implementation strategies to identify individuals at risk to allow preventive measures and early screening/diagnosis. Implementation of such measures is disturbed by improper use of the data, with breach of data protection as one of the risks to be heavily controlled. Population screening requires in depth cost-benefit analysis to justify healthcare costs, and the parameters screened should provide information that allow an actionable and deliverable solution, for better healthcare provision.

## Introduction

A new paradigm shift in cancer prevention and treatment is occurring in terms of predictive, preventive, and personalized medicine (PPPM) [1]. A patient will participate in, and be responsible for, more aspects of their healthcare. During the development of PPPM, many rapid, specific, and sensitive new methods for early detection of cancer will lead to a more efficacious and less onerous management of the patient, with better quality of life. This paper aims to develop the concept, principle, strategy, and technique for PPPM in cancer.

An important goal is to accurately diagnose, as soon as possible, a cancer so that effective treatment can be initiated to improve odds of recovery and to improve quality of life. Currently, healthcare systems utilize screening programs that incorporate genetic testing to promote early diagnosis and assess risk to genetic disease in an effort to prevent illness and hence reduce the burden on healthcare systems. Screening programs identify susceptible individuals/families eligible for preventive measures and recruit patients for treatment at early stages of the disease, as exemplified by BRCA1/2 screening. Screening programs aim to reduce recruitment of patients at advanced stage of the disease in order to enhance the effectiveness of healthcare.

In addition to preventive genetics, early detection and monitoring of resistance to therapy, genotype-guided prescription, and dosing provides information to improve tolerability of anticancer treatments. Adverse drug reactions and death by toxicity of chemotherapy is a catastrophic event that might, in a substantial proportion of patients, be prevented [2].

### Cancer and public health

#### Cancer incidence worldwide

**Figure 1:**
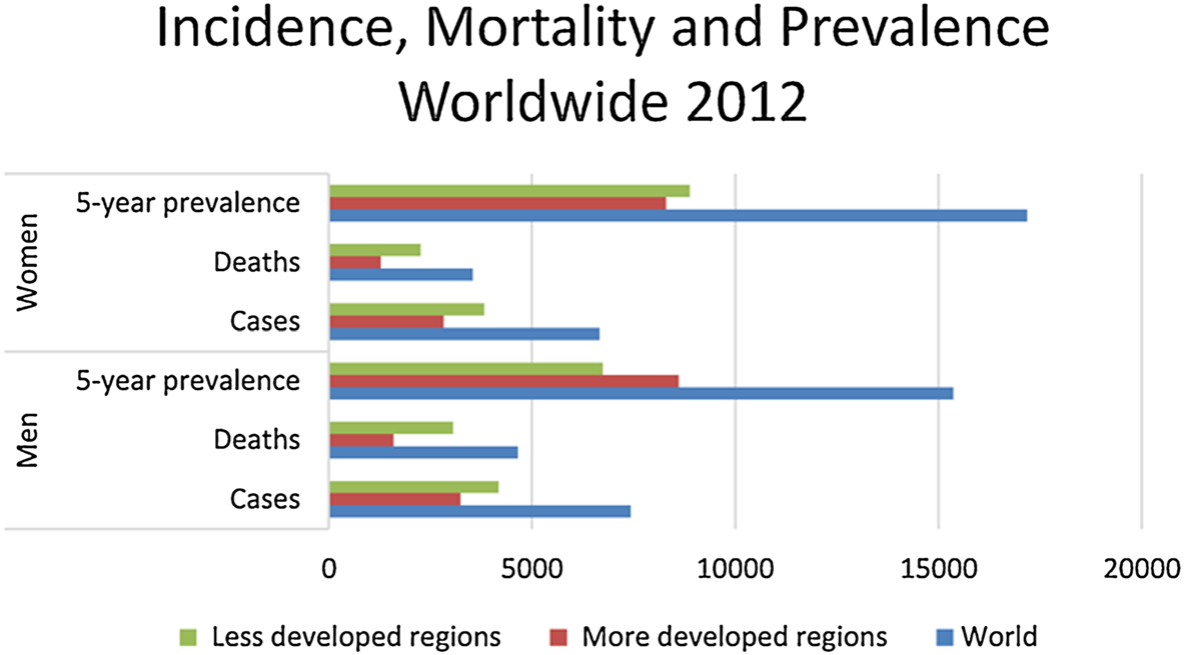
Worldwide incidence, mortality and 5 year prevalence in 2012. Data taken from GLOBOCAN [390] and prevalence data from [391].

The burden of cancer on public health is significantly high, with more than 14 million new cases worldwide, 8.4 million deaths, and a 5-year cancer prevalence of more than 35 million in 2012 (Figure 1). Considering a worldwide population of 7 billion in 2012, the prevalence percent is 0.5. Although all cancers are, of course, extremely important, focus is needed. The most frequently occurring forms of cancer in the EU are colorectal, breast, prostate, and lung cancers. Figure 1 shows the incidence, mortality, and prevalence in 2012 by gender. Breast cancer is ranked as the most prevalent cancer in most countries globally, as indicated by the higher prevalence of cancer in women (Figure 1). In general, the numbers of deaths follow the numbers of cases, when comparing developed and less-developed regions. Of interest, the 5-year cancer prevalence is higher in men from developed regions, most probably reflecting the significant decrease in mortality in prostate cancer.

#### The healthcare burden of non-communicable diseases

**Figure 2:**
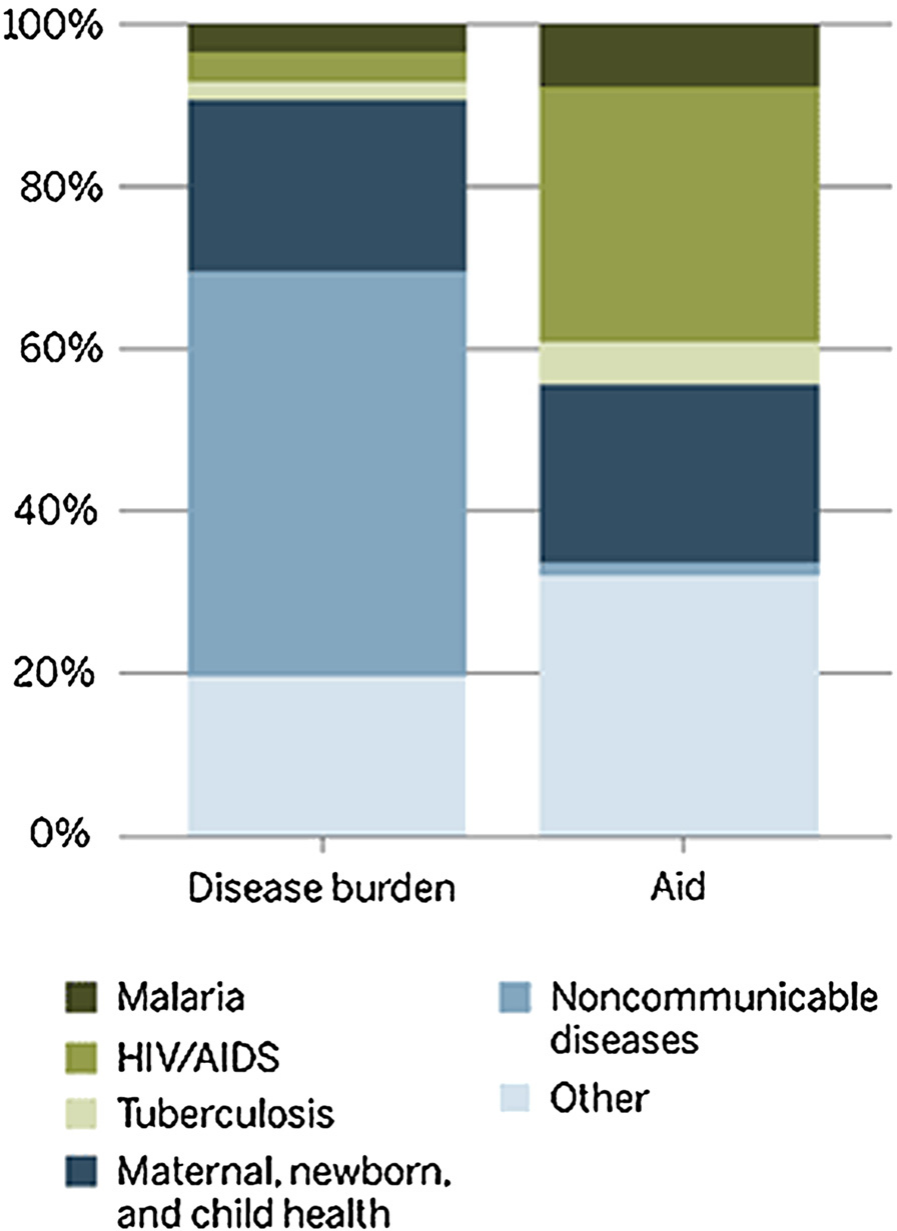
Disease burden versus aid (adapted from [4]).

Non-communicable diseases (NCDs), or chronic diseases, are defined as non-transmissible diseases and include cancer, cardiovascular disease, respiratory disease, and diabetes. NCDs cause 36 million deaths globally, and of the four main NCDs, cancer is the second most common cause of death (at 7.9 million per year), almost a quarter of the total. In addition, NCDs account for 80% of deaths in low-to-moderate income countries [3]. Studies on the economic burden of healthcare (as measured by disability-adjusted life years (DALYs)) have shown that, while NCDs comprise 45% of the DALYs, the international funding invested towards them has historically been disproportionately low, at less than 5% (Figure 2) [4].

In terms of a globally aging population, cancer is becoming a major burden on healthcare systems, and it has been established that various lifestyle factors are attributable to cancer, including physical inactivity, high-fat/low-fiber diets, obesity, smoking, and use or alcohol misuse [5]. It is known that one-third of cancers are preventable via addressing lifestyle risk factors. Therefore, multifaceted approaches are needed to address these issues, e.g., evidence-based strategies to lessen misuse of alcohol, among others [5].

Rapid increases in NCDs, such as cancer, are considered to be a threat to poverty-reduction initiatives in lower-income countries, where access to healthcare resources is limited and paying for healthcare pushes poor families further into debt. As a consequence, the global cancer burden (GCB) is expected to rise significantly and will disproportionately affect the less-developed world (LDW). In 2011, The United Nations General Assembly convened a high-level meeting on NCDs to look at their impact globally, in order to address this healthcare problem [6]. This meeting by the UN identified three priority areas for the 193 Member States to take. These included (1) surveillance and monitoring, (2) reduction of risk factors (e.g., tobacco and alcohol misuse, unhealthy diets and physical inactivity), and (3) a healthcare element in order to address those currently suffering with NCDs. Although it should be noted that some initiatives may eventually go beyond the remit of the general healthcare sector. For example, in order to diminish NCDs, engagement with sectors such as finance, industry, and commerce will be necessary.

Recent work by Love et al. (2010) highlights the complexity of healthcare delivery for oncology and cites that the multiple challenges are entrenched in socio-economic, political, and public health issues [7]. Moreover, the International Cancer Expert Corps (ICEC) have recently highlighted the (currently limited) availability of training and career opportunities in global health [8], while also highlighting the ethical aspects of addressing healthcare for low-to-middle income nations. Given the complexity of (a) addressing cancer as a globally increasing NCD and (b) ethically apportioning healthcare investment, it seems that a holistic approach is necessary in which policymakers and science/medical innovators should combine strategies to address this socio-economic challenge.

Moreover, the implicit economic costs of personalized medicine (e.g., targeted cancer therapy using molecular profiling) will also prove to be a challenge to healthcare providers worldwide. In order to address these challenges, international collaborations will be essential to determine the best and most cost-effective use of targeted therapies. These issues are under discussion by numerous studies, including Arnedos et al. (2014), who recently cited the need for “new organizational and medico-economics approaches” to reduce the financial burden presented by personalized medicine [9].

Worldwide, an increase in the yearly incidence of cancer occurs, from the current 14 million to an estimated 22.2 million by 2030 [10]. Of this figure, 13.1 million deaths have been predicted. An interesting perspective has been added to the debate by Gulley and Morgan (2014) who cite how the high cancer prevalence and mortality prevalent in developing nations might be addressed more efficiently [11]. For instance, because almost 25% of these cancers are infection-related, they postulate that the increased use of molecular assays provide a cost-effective method to detect relevant pathogen genomes [11]. Moreover, previous successful business models, such as the Yunus’ social business model [12], might also highlight business prototypes that could be applicable to oncology healthcare activities. Although beyond the remit of this current paper, they indicated the wide range of healthcare topics and challenges that will need to be addressed in the near future [13,14].

Clearly, reducing the socio-economic burden of cancer on global healthcare systems is a complex and multifaceted challenge [15,16]. Beyond the strategies of targeted therapies and pharmaco-genomic analyses (discussed below), other factors must be considered, such as preventative schemes addressing lifestyle-based risk factors (obesity, inactivity, smoking, and alcohol misuse). Moreover, as lower-income nations are disproportionately affected by cancer (as an NCD), there are also wider ethical issues in reducing the ensuing healthcare inequalities that will be presented.

#### Cancer and gender

Life-long risk factors for cancer development is influenced by gender-related factors such as different perception to healthy diets, gender differences in lifestyle, susceptibility to different infectious agents, hormonal levels, different metabolism rates, and the use of different medications, exemplified by the increased risk of breast cancer in women using contraceptive pills and hormone replacement therapy.

Of interest, there are also differences in bioavailability of drugs in females and males due to gender-specific variations in expression and genotype distribution of various drug-metabolizing enzymes (DME) [17]. Significant gender differences were reported in the occurrence of adverse effects of non-steroidal anti-inflammatory drug diclofenac (DCF) with increased risk in males for liver fibrosis and hepatocellular carcinoma [18] and adverse effects on the cancer chemotherapeutic drug, ifosfamide, associated with a more rapid CYP3A4-catalysis, suggesting a higher susceptibility to neurotoxic side effects in women [19]. In addition to gender-associated genotypic differences in DME that increase risk to cancer development and predispose individuals to drug-related side effects, expression and activity of DMEs are also under the effect of dietary factors [20], exposure to drugs [21], disease states [22], pregnancy [23], and endogenous hormonal factors [24]. There is a significant difference in these factors between males and females [25,26].

The regulation of DMEs by sex-related hormones is exemplified by the regulation of the cytochrome P450 oxidase CYP27A1 activity and expression in the presence of estrogen [24]. CYP27A1 converts cholesterol to its primary metabolite 27-hydroxycholesterol (27HC), which increased ER-dependent growth in breast cancer mouse models. Hence, interference with the metabolism of cholesterol and lowering circulating cholesterol reduces the risk to develop breast cancers and enhance response to hormone therapy [27].

#### Implementation of screening programs

Screening programs aim to identify individuals with a genetic predisposition to cancer eligible for preventive measures, to recruit patients at an early stage of the disease to allow effective treatments, and to identify individuals who harbor high-risk agents for cancer initiation eligible for treatment to eliminate the high risk factor, such as viruses. Various markers are used today as part of the public health initiatives to ensure (1) early diagnosis, exemplified by PSA testing; (2) characterization of monogenic syndromes that lead to cancer initiation, such as the BRCA1/2 [28] and hMSH2 [29] genes contributing significantly to an increased risk of breast and hereditary nonpolyposis colorectal cancer (HNPCC), respectively, and (3) genetic subtyping of human papillomaviruses (HPVs) to identify high-risk individuals to develop cervical cancer. Techniques to identify tumor-related markers using noninvasive sampling, will provide the proper tools for screening programs [30,31]. Screening population at risk requires in depth cost-benefit analysis to justify the healthcare costs. Mass screening of the entire female population for BRCA1 mutations is not justified within a public healthcare system due to the low incidence of the mutations in a population, as opposed to the high penetrance of BRCA1 mutants in individuals with family history. Hence, screening of familial cases is more than justified when the right tools and infrastructure to implement programs are utilized.

### Risk factors predisposing to cancer progression

#### Viruses and cancer risk

Increasing epidemiological evidence provides information on infective etiology of specific cancers. Prevention of infections is a healthcare measure to reduce the incidence of human cancers worldwide. Various viruses are now recognized to be a major risk factor in cancers, including the sexually transmitted HPVs in cervical lesions and cancers, hepatitis B virus in hepatocellular carcinoma, and Epstein-Barr virus associated with various lymphoid malignancies. It is also recognized that retroviruses are a major contributor to the infective etiology of childhood leukemia.

Infection of epithelial cells with HPV is a major risk factor for cervical intraepithelial neoplasia (CIN) and cervical carcinoma [32]. In Europe, 328 million women (age ≥15 years) are at risk to develop cervical cancer. Cervical cancer ranks the fifth most frequent cancer among women with 58,000 diagnosed annually [33]. The early age of onset of cervical cancer ranks the disease the second most common female cancer in women aged 15 to 44 years in Europe.

Because current vaccines against HPV 16 and 18 are not designed to protect against other high-risk (HR)-HPV genotypes [34], epidemiological studies within populations are important to estimate the impact of the use of vaccines on the healthcare system. HPV infections are also associated with other anogenital cancers, head and neck cancers, and other cancers, including the role of HPV in the development of breast cancer [35].

#### Human papillomavirus-induced cervical precancerous lesions

Today, it is known that in the development of HPV-induced cervical dysplastic lesions, the key impact has a specific cellular immune reactivity to HPV, especially the Th1-type response of T helper cells, that develops by balancing various opposition groups of cytokine, in particular, pro- and anti-inflammatory cytokines [36]. The importance of the immune reactivity is confirmed by the fact that patients with immunodeficiency states with suppressed cellular immunity, such as AIDS, are extremely susceptible to HPV-induced cancers. It is known that immune suppression influenced oncoproteins E6 and E7 of HPV high-risk oncogenic gene expression of interferon, interferon genes, and IL-18 production as well as increased production of immunosuppressive cytokines that inhibit the development of T helper cells of Th1-type and the production of interferon-γ and IL-2, play a key role in the evolution of mechanisms against HPV immune factors [36]. The presence of low-avidity IgG antibodies to HSV-1 and/or HSV-2 in the serum of patients with HPV-induced cervix precancerous diseases does not depend on the reactivation of HSV genome (genital herpes or generalized herpetic infection), which might be an indication for treatment as immunomodulators and antiviral (antiherpetic) drugs.

Induction of oxidative stress promotes HPV-initiated carcinogenesis. In inflammation, reactive oxygen species (ROS) and nitric oxide (NO), generated by inflammatory cells, play a key role in carcinogenesis. Thus, ROS can induce the formation of 8-oxodG, an indicator of oxidative DNA damage whereas NO can induce the formation of 8-nitroguanine, a marker of nitrative DNA damage. These factors are potentially mutagenic, which might account for the cancer-promoting effect of inflammation. It is reported that high-risk HPV types promote inducible NO synthase-dependent DNA damage, which leads to dysplastic changes and carcinogenesis [37]. Whereas therapeutic treatments cannot be based exclusively on the abatement of oxidative stress, neutralizing this cellular disorder could minimize collateral damages associated with the transformation of biomolecules in the cytosol. There were illustrated extensive interrelations among virus action, cellular oxidative stress, gene damage, multiple immune pathways and proteomic changes in diabetes mellitus, cancer, and many chronic disorders development, many of them were also related to HPV infection [38].

#### Gut microbiota, inflammation and cancer

Metabolic disturbances are associated with a number of diseases, including cancer development in lung, breast, uterus and ovary, and others such as cardiovascular diseases, violation of ovarian-menstrual cycle, local and systemic immunity, and dyslipidemia. Cancer and coronary heart disease are the most important disorders that cause alarming mortality and morbidity in humans [39]. For many years, atherosclerosis and cancer were considered to have a completely unrelated pathogenesis and disease progression pathway featuring separate therapeutic strategies. The various predictive and etiological factors, biomarkers, and molecular pathways of disease development and progression that are common to atherosclerosis and cancer suggest that the two most common diseases worldwide are far more closely aligned than previously believed. Both diseases share common etiological factors: genetic predisposition, age, sex hormones, cigarette smoking, high dietary fat intake, toxins, and mutagens. The consequences of the abovementioned etiological factor actions are cell cycle deregulation, oxidative stress, chronic inflammation, endothelial dysfunction, dysregulation of apoptosis, and angiogenesis, DNA instability, and impaired DNA repair. In addition to the aforementioned biological markers and pathogenic factors common for atherosclerosis and cancer disease development, a number of additional genetic pathways have been implicated in the progression of both diseases. The TGF-ß signaling pathway, other growth factors, cell adhesion molecules, the Wnt-ß-catenin signaling pathway, excess matrix digestion associated with matrix metalloproteases, and NF-kB signaling pathway represent other common molecular progression pathways shared by both diseases. Shared disease progression in atherosclerosis and cancer is the emergence of similar novel approaches to therapy [40,41].

Signals from the intestinal microbiota are important for normal host physiology; alteration of the microbiota (dysbiosis) is associated with multiple disease states [42]. Identification of components of the microbiota and elucidation of the molecular mechanisms of their action to induce pathological changes or exert beneficial, disease-protective activities could aid in our ability to influence the composition of the microbiota and to find bacterial strains and components (e.g., probiotics and prebiotics) whose administration might aid in disease prevention and treatment. Examination of the role of the microbiota in human illnesses using animal models of human diseases reared in defined conditions could allow insight into the unusual complexity of the mechanisms involved in the initiation and maintenance of chronic diseases [43].

Current knowledge shows that the colonic microflora is involved in the etiology of colorectal cancer. Intestinal bacteria can produce substances from dietary components that have genotoxic, carcinogenic, and tumor-promoting activities. The bacterial groups (lactobacilli and bifidobacteria) have much lower activities of enzymes that can generate carcinogens than do other gut microflora components such as clostridia and bacteroides. The balance of microbial types in the gut is very important and beneficial modulation of the intestinal microflora could decrease the colorectal cancer risk [44]. It might be hypothesized that not only common approaches to therapy but also common preventive strategies could be efficacious in both diseases. Probiotics and substances of natural origin can represent such a preventive approach.

#### Cancer predisposing syndromes

Inherited mutations in the genes phosphatase and tensin (PTEN), p53, STK11/LKB1, hMSH2, RB1, MEN1 and VHL give rise to predisposing syndromes, namely Cowden’s disease [45], Li-Fraumeni syndrome [46], Peutz-Jeghers syndrome [47], Lynch syndrome [48], retinoblastoma [49], Wermer syndrome [50], and von Hipple-Lindau syndrome [51], respectively. The estimated lifetime risk (70 years) for (1) the development of breast cancer is 85.2% (71.4%–99.1%) in individuals with Cowden syndrome and 45% (27%–68%) in individuals with Peutz-Jeghers syndrome; (2) the development of colorectal cancer is 48% (30%–77%) in individuals with mutations in hMSH2, one of the genes involved in Lynch syndrome, and 9% (3.8%–14.1%) in individuals with Cowden syndrome.

These inherited susceptibility genes have a low frequency, with a high penetrance. Hence, these genes are candidates for a testing program in specialized clinics to screen family members of patients, identify risk, and initiate preventive monitoring, or discuss possible clinical solutions to reduce the risk significantly [52]. Screening programs for cancer susceptibility genes is exemplified by screening of BRCA1/2 in families of breast cancer.

### Understanding cancer at a molecular level

A personalized therapeutic plan is important for effective individual patient care. Thus, many clinicians and basic scientists have sought to develop better diagnostic tests to predict patients’ responses to chemo, radiation, and targeted therapy [53,54]. However, cancer heterogeneity and individual differences make it difficult to develop effective cancer treatments [55,56]. Our understanding of the molecular mechanisms of cancer has to progress further to accelerate the development not only of more therapeutic drugs but also more sophisticated diagnostics for personalized treatment [57,58]. Finally, personalized treatment will be practical in the near future, with optimized prescriptions to optimize the right drug to the right patient at the right dose at the right time for the right duration.

Here, we briefly review the characteristics of cancers at various molecular levels, at the gene, protein, and metabolite level, as well as the current status of molecular cancer diagnostics and preventative markers.

#### At the gene level

Genetic aberrations play a significant role in cancer development [59]. Alterations in genomic sequences occur in the initial stage of carcinogenesis. Thus, the discovery of genetic alterations that occur in cancer development has been used to detect cancers [60-62]. Such signature changes include single-nucleotide polymorphisms (SNPs), the number of gene copies, microsatellites, and promoter methylation status.

#### SNPs

SNPs are the most common genetic variations and occur approximately every 1,000–2,000 bases in the human genome [63,64]. Most human sequence variation is attributable to SNPs, with the rest due to insertions or deletions of one or more bases, repeat-length polymorphisms, and rearrangements. When SNPs are in important genes, allelic imbalances are induced and, therefore, carcinogenesis might be initiated. SNPs are a promising tool to identify patterns of cancer risk. SNPs are detected by the profiling of transcription patterns. The frequency of shared polymorphisms might provide information on relative rates of mutation, recombination, and gene conversion [65]. For example, it has been known that protein phosphatases (PPs) might function as tumor suppressors by antagonizing protein kinases [66]. It was found that genetic mutations (two missense mutations) in the regulatory subunit 3 of the PP1 (PPP1R3) gene function as a tumor suppressor in human carcinogenesis, wherein five SNPs were identified in two colorectal carcinomas and ovarian carcinoma [67]. Hence, variable genetic polymorphisms in the PPP1R3 gene can be involved in human carcinogenesis.

To assess the susceptibility of individuals with genetic variants to cancer, cumulative evaluation of all SNPs within an individual is required. For example, common variants on human chromosome 8q24 were identified to be associated with prostate cancer susceptibility [68]. A genome-wide association study of SNPs in prostate cancer patients showed rs6983267 in the centromeric locus association with prostate cancer [69]. Additionally, another study reported that a genome-wide association scan of tagSNPs in colorectal cancer patients found that common, low-penetrance susceptibility alleles at 8q24.21 predispose to colorectal cancer development [70]. Furthermore, the most representative colorectal adenoma-associated SNP was rs6983267, also at 8q24.21, which causes enhanced Wnt signaling [71].

Accumulated SNP data can accelerate the use of SNPs for detection of cancers. Analysis of SNPs has been performed by hybridization of DNA probes or high-throughput technologies to identify cancer-associated polymorphic alleles [65,72,73].

#### Aberrant copy number

Differences in gene-copy number are another highly significant type of genetic variation. All cells have two copies of autosomal genes. When a mutation occurs in one allele of a tumor-suppressor gene, loss of heterozygosity (LOH) can lead to carcinogenesis. Because variation in gene copy number can affect the level of protein expression, copy-number variation is one of the most important determinants of individual traits between patients, including differences in susceptibility to various cancers.

Proofreading and DNA mismatch repair (MMR) are integral parts of the maintenance of high-fidelity DNA replication [74]. Defects in MMR increase overall genetic alterations, which cause cancer incidence. A somatic P286R substitution in the conserved exonuclease I domain of DNA polymerase ε was found in 52 sporadic colorectal tumor specimens. LOH of the variant allele (P286R mutation) functioned as a strong factor to elevate cancer incidence [75].

LOH can be detected by various approaches, based on PCR, in most pre-neoplastic lesions and primary tumors. Whole-genome sequencing has the potential to capture targeted coding sequences with high sensitivity and specificity for the detection of homozygous and heterozygous variants [76]. Thus, whole-exome sequencing might have clinical utility in the discovery of cancer-associated LOH and in making clinical diagnoses [77].

#### Microsatellite alterations

Microsatellite instability (MIN) involves changes in thousands of microsatellite sequences scattered throughout the genome. Microsatellites are simple sequence repeats (SSRs) or simple/short tandem repeats in which the repeats contain 1–13 base pairs [78]. SSRs affect the genes with which they are associated. Thus, changes in the numbers of SSRs can affect gene regulation, transcription, and protein function [79]. For example, one report demonstrated that microsatellite instability at (CA)_n_ repeats on human chromosomes 5q, 15q, 17p, and 18q correlated significantly with proximal colon cancer and affected patient survival [80].

Instability at the nucleotide level is the result of mistakes in DNA repair, which can lead to tumor progression and tumor heterogeneity. In HNPCC, mutations in the *hMSH6* gene are caused by post-replicative mismatch repair. This mutation has been studied with analysis of the pattern of the polymorphisms, which could allow better diagnosis of HNPCC [81]. Additionally, the alteration of microsatellite loci causes changes in the length of repeats during replication as a result of dysregulation in proofreading by exonuclease and MMR enzyme reactions [81]. Moreover, somatic mutations of microsatellites have also been observed in cancers of the gastrointestinal tract [82], lung, soft tissue, breast [83], and bladder [84].

#### Promoter hypermethylation

DNA methylation is an important factor in many processes, including DNA repair, genome stability, and the regulation of chromatin structure [85,86]. DNA methylation refers to the covalent addition of a methyl group at the 5’-carbon of the cytosine ring, to form 5’-methylcytosine [87]. These methyl groups effectively inhibit transcription. “CpG islands” are local regions where CpG sites are found more frequently in small stretches of DNA. Gene silencing in cancer is associated with promoter hypermethylation [88].

DNA hypermethylation has been observed in various cancers. For example, BRCA1 activity is markedly decreased in invasive breast tumors. In breast cancer, however, hypermethylation is often found in *BRCA1* [89]. In hematopoietic malignancies, DNA hypermethylation often silences tumor suppressor genes that encode cell adhesion molecules and growth-regulatory proteins [90]. Methylation of the MLH1 MMR gene has also been observed in colorectal cancer [91]. Silencing of the von Hippel-Lindau (VHL) tumor-suppressor gene occurs by DNA methylation in renal carcinoma, similar to *BRCA1* in early breast cancer [90,92]. Moreover, methylation in the promoter of the *O*^*6*^*-methylguanine methyltransferase* (*MGMT*) gene is known as a clinical prognostic factor regarding the action of alkylating agents in gliomas [93]. MGMT is involved in DNA repair in colon cancer, lymphoma, non-small cell lung cancer, and brain tumors. Thus, its inactivation by promoter methylation may be important in the expected utility of targeted therapeutics [94].

Tremendous advances in high-throughput screening and detection, such as DNA microarrays and multiplexed assays, have allowed the identification of novel biomarkers. DNA microarrays offer rapid surveillance of expression patterns of tens of thousands of genes in a single experiment. DNA microarrays, as a tool for profiling transcription patterns, can analyze hundreds or thousands of genes in a single assay [95] and replace differential PCR-based approaches in some applications. Gene expression profiles can indicate transcriptional variation between healthy and cancerous cells. The use of this technique generally relies on clustering analysis of several genes that differentiate cancer versus normal profiles rather a single gene.

#### Messenger RNA level

Messenger RNAs (mRNAs) and microRNAs (miRNAs) have been a focus as key components in understanding cancer and are known to contribute to the regulation of cancer-signaling pathways [96]. Dysregulation at the mRNA level occurs in various cancers because the molecular processes of mRNA are complex and multilayered.

The most common method to identify and quantify mRNA levels from patients’ samples is reverse-transcription PCR (RT-PCR) [97]. For example, carcinoembryonic antigen (CEA) levels in colorectal cancer can be detected by RT-PCR. Increased CEA mRNA isolated from RNA in serum might indicate micrometastatic bone disease in patients with CEA-expressing carcinomas, using CEA-specific PCR [98]. Prostate-specific antigen (PSA) mRNA can also be detected and quantified with molecular assays that detect PSA-synthesizing cells in the peripheral circulation of patients with prostate cancer [99].

Changes in mRNA transcript stability have also been implicated in cancer progression. For example, destabilization of mRNAs by a protein that binds structural elements of mRNAs can regulate cancer progression [100].

Quantitative mRNA analysis can also be used to detect multiple biomarkers in the serum of patients with breast cancer [101]. In colon cancer, levels of mRNA encoding dihydropyrimidine dehydrogenase, thymidylate synthase, and thymidine phosphorylase have allowed predictions of responses to 5-fluorouracil therapy [102].

#### MicroRNA

Isolation and preparation of total RNA from tissues and/or body fluids such as blood or urine make it possible to globally analyze RNA profiles, including small RNAs, such as miRNAs. Recently, some reviews have focused on the role that deregulated mRNA translational control in eukaryotic cells and mammalian systems might play in carcinogenesis [103,104]. miRNAs are small non-coding RNAs involved in virtually all functional aspects of eukaryotic cells, including embryonic development, cell differentiation and proliferation, cell death, energy metabolism, and antiviral defense. Dysregulation of miRNAs is often associated with various cancers because the control of miRNA levels is important for normal functioning in cells [105,106]. miRNAs serve as key regulators of gene expression and affect cellular gene expression networks markedly [107,108]. It has also been shown that changes in the expression of miRNA might contribute to carcinogenesis.

Profiling the expression of miRNAs can allow human cancers to be classified according to the lineage and the state of the cancers. For example, miRNA profiling studies showed that distinct patterns of miRNA characterize different hematopoietic differentiation stages in acute leukemia samples: the *BCR-ABL*, *TEL-AML1 -*(acute myeloid leukemia 1 also known as runt-related transcription factor 1 (*RUNX1*)) and mixed lineage leukemia (*MLL*) rearrangements could be distinguished [109,110]. miRNA includes onco-miRNAs and tumor-suppressor-miRNAs. For example, miR-21 is aberrantly expressed in glioblastomas, and its overexpression might induce carcinogenesis by blocking expression of critical apoptosis-related genes [111,112]. In the human B-cell line P493-6, overexpressing MYC, the miR-17-92 locus has tumor suppressor activity. Its expression curbs MYC-involved proliferation by inhibiting the expression of E2F1 [113].

Because the dysregulated expression of miRNA genes has been attributed to carcinogenesis controlled by multiple oncogenes or tumor-suppressor genes, understanding miRNAs will be important to understanding the cancer too and provide crucial clues for the development of future miRNA-based therapies [114]. Advanced genomic technologies have now determined that at least 90% of the genome is actively transcribed [115,116].

#### At the protein level

Dysregulation in protein expression, post-translational modifications (PTMs), protein-protein interactions, as well as dysfunction in specific protein activity and inappropriate localization of proteins can all occur during carcinogenesis [117]. Abnormalities in the modification of proteins may be important for the signaling, metabolic, or structural properties of the cell and cause disorders in biogenesis, protein aggregation, cell metabolism, or signaling. Understanding such post-translation events is possible through recently developed proteomics-based tools.

#### Dysfunction in enzyme activity

Dysfunction in the catalytic activity of certain enzymes can lead to pathogenic alterations in the pattern of gene transcription within a particular cell type. For example, changes in protein methyltransferase (PMT) activity play critical roles in carcinogenesis [118]. Deregulation of PMT activity, affected by genetic alterations, can reduce levels of transcription of specific tumor suppressor genes or induce those of specific oncogenes [118,119]. Thus, several PMT enzymes might be targets in personalized treatment such as by using small-molecule inhibitors of PMTs (PMTis), including DOT1L and EZH2 [120].

#### Mislocalization of proteins

The appropriate localization of proteins is a fundamental requirement for them to exert their expected function. Protein mislocalization can lead to their inactivation (loss of function) or overexpression (gain of function) [121]. For example, a shift in BRCA1 cellular localization often occurs in human breast cancers of differentiated grade and patients with BRCA1 mutations [122-124]. Blockage of the nuclear localization of full-length BRCA1 was observed when a mutation was found in the carboxyl-terminal domain of the BRCA1 protein [125,126]. Thus, gene mutations and alternative splicing in the *BRCA1* gene might have effects on the nuclear import and distribution of the BRCA1 protein, which might play a role in breast cancer development [127].

#### Protein kinases

Kinases have critical roles in phosphorylating molecules in signaling pathways. Deregulation of kinases can turn on or change signals governing proliferation, migration, survival, and differentiation. Oncogenic kinase proteins participate in various cellular signaling steps in malignant cells [128].

Tyrosine kinase signaling is a representative signaling pathway involved in cancer mechanisms. Tyrosine kinases, as a class of enzymes, are key regulators of many important cellular regulatory processes, such as cell growth, differentiation, cell survival, cell migration, and cell-cycle control, that can contribute to cancer development and progression [129]. Tyrosine kinases include receptor protein kinases—e.g., epidermal growth factor receptor (EGFR) (ErbB/HER) family members, vascular endothelial growth factor receptors (VEGFR), and platelet-derived growth factor receptors (PDGFR) (α and β)—and non-receptor protein kinases, such as BCR-ABL and KIT [130]. Their mutations contribute to deregulation of tyrosine phosphorylation, which can play an important role in oncogenesis. In non-small cell lung cancer, somatic mutations of EGFR2 have been documented [131]. Also, somatic mutations of EGFR2 and EGFR3 have been observed in human bladder and cervical carcinomas [132,133]. Aberrant expression of ErbB receptor also causes the development of epithelial cancers [134].

#### Other proteins

The most well-known protein-based marker is likely to be PSA, which is found at high levels in the serum of patients with prostate cancer [135]. Cancer antigen (CA)125, another older marker, is widely used to detect ovarian cancer [136]. Furthermore, upregulated fecal tumor M2-protein kinase (M2-PK) has been observed in the stool of colorectal cancer (CRC) patients [137,138].

Recent advances, especially in mass spectrometry, have enabled high-content quantitative information about patient samples and facilitated the analysis and functional characterization of protein complexes and protein pathways [139,140]. Further genomics and proteomics technologies in development have further promise to identify new biomarkers, which might facilitate predictions of cancer development/progression and to enhance personalized medicine.

#### Metabolites

Metabolites are measured systemically to assess the dynamics of changes in metabolites and the metabolome in a cell or tissues, as associated with various physiological and pathological states in a patient [141]. Analysis of metabolites can allow a global understanding of discrepancies in biological systems in individuals. Moreover, recently developed high-throughput technologies can provide researchers with a deeper understanding of cancer-specific metabolism and facilitate multidisciplinary approaches to the study of cancers [142].

The metabolites used as cancer markers can be classified with their associated biochemical pathways. Many of the metabolic disturbances observed are due to the need to support the cancer’s high cell growth and proliferation, such as changes in glycolysis, the tricarboxylic acid (TCA) cycle, transcription and translation, and purine and pyrimidine metabolism.

#### Glycolysis

Because of the Warburg effect, glycolytic activity is increased in cancer cells. Thus, adenosine triphosphate (ATP) is produced in large amounts via the glycolysis pathway in most cancers [143]. Additionally, the expression of Na^+^-dependent glucose transporters (GLUT) is increased when glycolytic enzymes, such as phosphoglycerate kinase 1 and alpha-enolase are activated [144,145]. The decrease in glucose, as a result of rapid cancer cell proliferation, can be measured to diagnose hepatocellular carcinoma [146], renal cell carcinoma [147], and cervical cancer [148]. Together with changes in glucose, elevated levels of pyruvate and lactate can also serve as tools to differentiate normal tissue from hepatocellular carcinoma [149], colorectal cancer [150], stomach cancer [151], and oral cancer [152].

#### Tricarboxylic acid cycle

Alterations in the TCA cycle, such as lower levels of citrate and elevated levels of fumarate, malate, and succinate, are found in various cancers. For example, functional impairment of TCA cycle results from mutation of fumarase gene in leiomyoma [153].

In gliomas, mutations in the enzyme cytosolic isocitrate dehydrogenase 1 (IDH1) can cause loss of the enzyme’s ability to catalyze the NADPH-dependent reduction of α-ketoglutarate to 2-hydroxyglutarate (2HG). Overaccumulation of 2HG has been detected in gliomas [154].

#### Pyruvate metabolism

When levels of pyruvate oxidation become lower and glycolysis is activated, levels of lactate and alanine become elevated. This metabolic phenotype can provide a selective growth advantage for cancers [155,156]. Elevation of pyruvate contributes to the formation of hypoxic conditions in cancer cells and might ultimately induce cancer development and progression [156]. Additionally, in several gliomas, markedly increased alanine levels have been detected [157].

#### Other metabolic markers

Cancer metabolic markers related to brain and prostate cancers, such as *N*-acetyl-aspartate (NAA) and sarcosine, respectively, have also been identified and assessed [99,158,159].

We have briefly reviewed the molecular traits and mechanisms of cancer at each molecular level. In the initial stages of cancer development, changes in SNPs, the number of gene copies, microsatellites, and promoter methylation have been shown to be major events in carcinogenesis. With respect to the expression of many genes, genetic alterations at the RNA level have also been regarded as hallmarks of cancers. miRNAs have received much attention because of their role to induce translational inhibition by silencing targeted mRNAs related to cancer metabolism. Furthermore, cancer-specific features at the protein level involve altered protein expression and protein-protein interactions, modifications of protein structure and enzyme activity, and perturbations of cancer-related pathways and localization during carcinogenesis. Metabolites have recently been highlighted as biomolecules that indicate aspects of cancer metabolism, such as changes in glycolysis, as well as provide significant information on the expression of cancer phenotypes.

Although considerable knowledge about the molecular bases of cancer has been accumulated thus far, we still have many unsolved problems in cancer treatment. To increase our understanding of cancer and to develop truly personalized medicine, further advances in high-throughput “omics” technologies are needed. These technologies will allow global interpretation of discrepancies in the biological systems of individuals and will facilitate multidisciplinary approaches to personalized medicine in the near future.

### Current predictive markers

#### Colon cancer

CEA levels have been commonly used in clinical practice as part of the follow-up after curative resection for CRC [160-162].

5-Flurouracil (5-FU), an antimetabolite, is the mainstay of all current standard CRC chemotherapy regimens. Deficiency in dihydropyridimine dehydrogenase (DPD) has been associated with increased 5-FU toxicity through reduced metabolism to result in accumulation of 5-FU and, hence, more serious and life-threatening side effects [163,164]. The common DPYD gene (the DPYD gene locus) can predict the accuracy of screening CRC patients before administering 5-FU [165].

Evaluations of the benefits of adjuvant 5-FU chemotherapy in mismatch repair-deficient and mismatch repair-proficient patients have been made. For mismatch repair-deficient patients, there was no statistically significant difference in relapse-free survival (RFS) or overall survival (OS) regardless of whether patients received 5-FU-based chemotherapy [166]. Also, there was no significant effect on survival or disease-free survival (DFS) of adjuvant 5-FU chemotherapy in 545 mismatch repair-deficient patients [167,168]. However, recently, CRC with a defective mismatch repair system did not respond to adjuvant 5-FU chemotherapy [169].

A mutant Ki-ras2 Kirsten rat sarcoma viral oncogene homologue gene (KRAS) accounted for ~ one-third of patients who did not respond to anti-EGFR therapy. Therefore, additional biomarkers for the efficacy of EGFR therapy are required [170]. Because B-RAF is the major effector of KRAS, a B-RAF mutation might well be useful to predict the response to anti-EGFR therapy for cancers in patients with wild-type KRAS, as well as analysis of the KRAS mutation [171].

KRAS remains the only validated predictive marker for anti-EGFR monoclonal antibody therapies (such as cetuximab or panitumumab) for CRC. Among the clinical studies that report positive outcomes, the CRYSTAL, OPUS, and PRIME trials were early representative trials with clinical outcomes.

Cetuximab Combined with Irinotecan in First-line Therapy for Metastatic Colorectal Cancer (CRYSTAL) was performed as a multicenter phase 3 trial [172]. When treated with irinotecan, fluorouracil, and leucovorin (FOLFIRI) plus cetuximab, there was a lower metastatic risk of metastatic CRC (mCRC) than with treatment with FOLFIRI alone. A limited efficacy of cetuximab was seen in KRAS wild-type tumors.

The Oxaliplatin and Cetuximab in First-Line Treatment of Metastatic Colorectal Cancer (OPUS) trials compared treatments of oxaliplatin/5-fluorouracil/folinic acid (FOLFOX-4) plus cetuximab vs FOLFOX-4 alone. Administration of cetuximab in combination with FOLFOX-4 in KRAS wild-type mCRC improved progression-free survival (PFS), response rate (RR), and overall response rate (ORR) [173].

The Panitumumab Randomized Trial in Combination with Chemotherapy for Metastatic Colorectal Cancer to Determine Efficacy (PRIME) examined the efficacy and safety of panitumumab plus infusional FOLFOX4 vs FOLFOX4 alone as an initial treatment for mCRC. Panitumumab-FOLFOX4 treatment significantly improved the PFS in patients with KRAS wild-type tumors [174]. In short, the KRAS mutation status influenced outcomes and was an effective predictive biomarker in CRC.

In initial studies, the KRAS mutation in codons 12 and 13 of exon 2 were analyzed to predict the outcomes of anti-EGFR monoclonal therapies [175,176]. Recently, because mutations of RAS have been established as predictive factors for anti-EGFR monoclonal antibody treatment, the treatment has also been more effective in patients with all RAS wild-type tumors rather than just those with a RAS mutation [177]. Moreover, in the American Society of Clinical Oncology (ASCO) 2014, one clinical study reported that mutations in RAS genes (KRAS/NRAS exons 2,3, and 4) predicted a lack of response to panitumumab plus FOLFIRI therapy for mCRC [178]. The 2014 National Comprehensive Cancer Network (NCCN) guidelines expanded the extent of the therapeutic standards for mCRC from KRAS mutations to all RAS mutational analyses, and the clinical use of EGFR-directed monoclonal antibodies cetuximab and panitumumab were allowed only in patients with wild-type RAS [179].

BRAF is one of the most common negative prognostic markers validated to date even though it is unclear that BRAF could play a role in the predictive marker in anti-EGFR therapy. It is a poor prognostic marker not only for the metastatic stage but also for stages II and III. For example, wild-type BRAF in mCRC appeared to show good PFS and OS outcomes in response to panitumumab and cetuximab, respectively [180]. Furthermore, in the MRC COIN trial, patients with advanced CRC who had a mutated BRAF showed a poor prognosis in response to the addition of cetuximab to oxaliplatin-based first-line combination chemotherapies [181]. In cetuximab plus FOLFIRI as a first-line treatment for mCRC, the BRAF tumor mutation was a strong indicator of a poor prognosis [182].

#### Gastric cancer

There are fewer effective targeted therapies for the clinical treatment of gastric cancer than there are for other cancers [183]. Trastuzumab (a recombinant monoclonal antibody against human epidermal growth factor receptor 2 (HER2)) was reported as a successful HER2-targeted therapy as a first-line treatment for HER2-positive advanced gastric or gastro-esophageal junction cancers [184].

In gastric cancer, HER2 is currently the only validated predictive marker for HER2-targeted agents, with other markers still to be validated [185]. Although amplification of MET is a good candidate as a predictive marker, further verification of its predictive value is needed. Immunohistochemistry of EGFR could not predict the clinical responses to cetuximab or EGFR tyrosine kinase inhibitors (TKIs) [186,187]. Although a mammalian target of rapamycin (mTOR) inhibitor could not improve the OS in a phase III trial (GRANITE-1), multiple phosphorylation sites of S6 (Ser 240/244 and Ser 235/236) could be correlated with improved disease control [188]. However, these still remain to be validated. Moreover, cisplatin and fluorouracil improved the PFS in low expressions of EGFR, FGFR2, and MYC [189].

#### Breast cancer

Great efforts have been made at the transcriptome level to discern which ER-positive early breast cancer patients would truly benefit from additional chemotherapy and who could be spared from excessive treatment and side effects [190]. Traditional classification systems of biological characteristics, hormone receptors (estrogen receptor (ER) and progesterone receptor (PR)) and HER2 (c-erbB2) status, might have limitations for patient-tailored treatment strategies.

The expression of the ER is the most important biomarker in breast cancer because it provides an index of sensitivity to endocrine treatments. Multiple clinical studies have demonstrated that ER-negative breast cancer patients are more likely to achieve a pathological complete response (pCR) with neoadjuvant chemotherapy, as compared to ER-positive patients, with pCR rates reported of 7%–8% vs 21%–33%, respectively [191,192].

The expression of the PR is strongly dependent on the presence of the ER. In rare cases of solely PR-expressing patients, some limited benefits from tamoxifen have been described; however, endocrine therapy is still widely recommended [193]. The Arimidex, Tamoxifen, Alone or in Combination (ATAC) Trialists’ Group published a report that suggest that patients with PR-negative breast cancer would obtain substantially greater benefits from anastrozole than from tamoxifen, as compared with PR-positive patients [194].

The HER2 oncogene was initially used as an indicator of patient prognosis. In cases of overexpression of HER2 (HER2-positive), breast cancer patients were more likely to suffer from relapses and tended to have a shorter OS. Through the development of the monoclonal antibody trastuzumab, which is targeted at HER2, the amplification status of HER2 became a highly predictive biomarker [195,196]. Recent studies also describe an association of HER2 amplification with benefits from adjuvant chemotherapies [197-199] as well as from combined chemotherapy in metastatic breast cancer [200-203].

Measurement of Ki67 has the potential to radically change the assessment of available prognostic markers. Findings from our group indicated that post-neoadjuvant chemotherapy measurement of Ki67 was a strong predictor for RFS and OS [204]. The role of cyclin E in the cell cycle suggests that increased levels might alter responses to chemotherapy and endocrine therapy. Altered levels of cyclin E increased the sensitivity of breast cancer cells to the effects of cisplatin and paclitaxel [205].

ERb mRNA levels were also used in the evaluation of patient prognoses. The availability of specific antibodies enabled ERb protein levels to be suggestive of a good prognosis, prolonged DFS, and a response to tamoxifen [206,207]. However, HER2-positive cancers, which showed amplification and overexpression of the ERBB2 gene, did not express hormone receptors and thus represented a poor prognosis [208-210].

HER-2/neu has been used in the clinical treatment of breast cancer since it gained FDA approval after immunohistochemistry testing. The American Society of Clinical Oncology and the College of American Pathologists (ASCO/CAP) also recommended use of the HER-2/neu test to select patients suitable for herceptin therapy [211].

Additionally, CA27.29, and CA15-3 are the most widely used serum biomarkers that are highly associated with breast cancer and are derived from the MUC1 gene [212].

Testing of the clinical activity of trastuzumab in women with HER2-positive breast cancer demonstrated that a single year of adjuvant chemotherapy treatment with trastuzumab improved the DFS [197]. Adjuvant chemotherapy (paclitaxel after doxorubicin and cyclophosphamide) with concurrent trastuzumab administrated to women after surgical resection of HER2-positive breast cancers had improved outcomes compared with adjuvant chemotherapy alone [198]. Although a nonanthracycline chemotherapy regimen combined with trastuzumab was related to cardiac toxicity, trastuzumab improved the survival rate in the adjuvant treatment of HER2-positive breast cancer. Therefore, for women with HER2-positive breast cancer, treatment with adjuvant trastuzumab improved the DFS and OS, and trastuzumab was recommended as an effective alternative to anthracycline-based regimens [199].

In HER2-overexpressed metastatic breast cancers, initial chemotherapy treatment with trastuzumab resulted in a longer time to disease progression, a longer duration of response, a lower rate of death at 1 year, longer survival, and a 20% reduction in the risk of death [200].

A randomized phase III study (TAnDEM) compared the clinical outcomes of anastrozol combined with trastuzumab to those of anastrozole alone in postmenopausal women with HER2/hormone receptor-positive metastatic breast cancer (MBC). Although there were more adverse and serious adverse events in the combination treatment of trastuzumab plus anastrozole than in the treatment of anastrozole alone, treatment of trastuzumab plus anastrozole significantly improved PFS, time to progression (TTP), clinic benefit rates (CBR), and ORR [213].

Cross-talk between the EGFR and the hormone receptor (HR) in HR-positive MBC results in resistance to conventional endocrine therapies [214]. To overcome resistance, lapatinib (a dual tyrosine kinase inhibitor) that blocks EGFR and HER2 was administered together with letrozole (an aromatase inhibitor). This led to enhanced PFS and clinical benefits in patients with HR/HER2 co-expressed MBC [215].

Trastuzumab emtansine (T-DM1) is an anticancer drug that has the anticancer effects of HER2-targeted trastuzumab and the cytotoxic activity of the microtubule-inhibitory agent DM1. Clinical effects were improved after administration of T-DM1 and of lapatinib plus capecitabine, as compared to treatment with trastuzumab plus taxane for patients with HER2-positive advanced breast cancer. In the T-DM1 group, PFS and OS both increased, and there was decreased toxicity [203].

#### Lung cancer

Cell cycle markers are some of the most powerful predictors of survival. In one study, multiple markers in 408 stage I patients were evaluated, and high Rb expression was associated with an improved 5-year survival rate, although the results did not achieve statistical significance [216]. Up-regulation of the cyclin D1 proto-oncogene is known to be important in the regulation of the cell cycle pathway. In a study limited to stages I and II, cyclin D1 expression was associated with shorter survival and the worst prognosis was observed in tumors with a combination of high cyclin D1 expression and loss of p16 expression [217].

Some mediators of apoptosis might be predictors of survival in lung cancer. For instance, as high expression of apoptosis inhibitors, survivin is significantly favorable for survival in patients with non-small-cell lung carcinoma (NSCLC), and up-regulation of Fas might also indicate a favorable prognosis in NSCLC with stage III disease [218,219]. Single studies of TNFR-1, TNFR-2, and TNF-a showed that these markers were also associated with improved survival rates in NSCLC patients [219,220]. In a study of 135 patients with stage I NSCLC, hypermethylation of the death-associated protein (DAP) kinase promoter was found in 44% of the tumors and was a significant independent factor that predicts poorer disease-specific survival [221]. Several other studies suggested that bcl-2 was an independent prognostic marker of improved survival [222-224]. In one small study of advanced disease, Bax expression was associated with improved median survival in stage IV NSCLC [225].

Other markers such as p53 and RAS have prognostic and predictive relevance in adjuvant chemotherapies (adjuvant cisplatin plus vinorelbine) in NSCLC. Protein overexpression of p53 was a significant prognostic marker of shortened survival as well as a predictive marker to indicate greater survival benefits of adjuvant chemotherapy in completely resected NSCLC patients [226]. Multifunctional proteins such as p53 and RAS are critical to cell cycle regulation, apoptosis, cell survival, gene transcription, response to stress, and DNA repair. Four large studies that were limited to stage I patients yielded conflicting results [216,227-229]. In the largest study, increased p53 expression had no effect on the outcome [228].

For p53, most studies focused on bcl-2 as a second marker. Two studies suggested that a combination of increased p53 expression and decreased bcl-2 expression indicated the poorest survival rates [230]. Similar results were also seen in a combination of p53 and Rb expression [231,232]. The interrelationships between these proteins in the cell cycle and the apoptotic pathways might explain the potentially synergistic effect of p53 and bcl-2 or Rb.

The mutational status of KRAS could also be a strong prognostic marker for adenocarcinoma of the lung. A point-mutation in codon 12 of KRAS was evaluated with RAS-specific DNA sequencing, which confirmed that the presence of a KRAS point-mutation in lung cancer indicated a poor prognosis and shorter DFS [233]. The KRAS mutational status was also indicative of a reduction in the therapeutic efficacy of EGFR-TKIs; however, it did not affect the chemotherapeutic efficacy. These recent findings demonstrated that mutations in EGFR or in KRAS affected the sensitivity to EGFR-TKIs, such as erlotinib. In NSCLC, there was a greater likelihood of response to chemotherapy when combined with erlotinib, as compared to chemotherapy alone. However, patients with KRAS-mutant NSCLC showed poorer clinical outcomes when treated with erlotinib and chemotherapy [234]. The presence of mutations in EGFR and KRAS could be a strong predictor of resistance to EGFR-targeted monoclonal antibodies.

In bronchioloalveolar carcinoma (BAC) and adenocarcinoma, a phase II trial to determine the efficacy of erlotinib against BAC subtypes was performed. Erlotinib proved active against BAC and adenocarcinomas and mixed subtypes of BAC. Evaluation of EGFR and KRAS mutations could predict the RR and PFS after erlotinib administration, and histologically enriched subsets derived from patients could help with clinical NSCLC trials of the use of EGFR-directed therapies [235].

In the Biomarker-integrated Approaches of Targeted Therapy for Lung Cancer Elimination (BATTLE) trial, erlotinib, vandetanib, erlotinib plus bexarotene, or sorafenib, were randomly used to treat chemorefractory NSCLC. For patients with mutant KRAS, treatment with sorafenib showed some impressive benefits [235].

Clinical outcomes of responses to gefitinib (an EGFR tyrosine kinase inhibitor) were higher in EGFR-mutated patients with advanced NSCLC. In addition, in the EGFR-mutated group, administration of gefitinib further increased PFS and the acceptable toxicity, as compared to standard chemotherapy [236,237].

Additional molecular markers might also be used to indicate tumor status. PDGF expression in the stromal cells might have a greater effect on angiogenesis and outcomes in NSCLC than tumor cell expression [238,239]. Basic fibroblast growth factor (bFGF) is released by proteolytic enzymes from the extracellular matrix, after which it increases the expression of other proteolytic molecules. In a follow-up study, there was an association between the presence of tumor-infiltrating macrophages and tumor IL-8 expression that suggest a mechanism for how macrophages adversely affected outcomes in NSCLC [240].

#### Liver cancer

Potential prognostic markers in liver cancer include p53 mutations, the PTEN homologue, c-met, c-myc, p18, p27, p57, serum VEGF, hypoxia-inducible factor-1 (HIF-1)a, MMP-2, MMP-7, and MMP-12, as well as proliferation indices, telomerase activity and aneuploidy.

DNA mutation analysis is a useful technique to assess p53 status. The majority of studies that investigate p53 mutations have found them to be associated with shorter DFS and OS [241-245]. Positive expression of the PTEN tumor suppressor gene has been identified as an independent prognostic factor for decreased OS following resection for hepatocellular carcinoma (HCC) [246,247]. In HCC, c-met is commonly over-expressed and has been associated with reduced OS in prognostic studies [248,249]. Expression of c-myc was increased in those patients who develop early recurrence post-hepatectomy [250], potentially through overexpression of cancerous inhibitor of protein phosphatase 2a (cip2a) [251].

Multivariate analyses have shown that Bcl-xL overexpression could independently predict decreased OS and DFS [252]. Garcia et al. found that expression of the pro-apoptotic protein Bax independently predicted increased OS following resection of HCC [253].

Survivin is a member of the inhibitor of apoptosis protein (IAP) family of anti-apoptotic proteins. Patients with tumors that express survivin mRNA suffered higher rates of recurrence and poorer disease-specific survival rates than those with tumors that did not express survivin mRNA [254,255]. A high survivin/GADPH mRNA ratio was shown to independently predict tumor recurrence after hepatectomy and was associated with reduced DFS [256].

HIF-1 is expressed by several human malignancies, and overexpression has been associated with resistance to chemotherapy and poor prognosis in some cases. OS and DFS were significantly improved in patients with low preoperative serum Il-8, as compared to those with higher levels [257]. Multivariate analyses showed that Il-8 was an independent prognostic factor for OS.

High expression of transforming growth factor beta1 (TGF-β1) is an independent prognostic factor for reduced survival in patients with inoperable HCC [258]. Elevated urinary TGF-β1 was prognostic of shortened survival in patients with HCC, although levels were measured at the time of diagnosis, and not all patients underwent surgery [259].

Microsatellites (small tandem repeats of DNA bases) are present throughout the genome. Microsatellite instability (MSI) is caused by mutations in DNA mismatch repair genes. Salvucci et al. found no association between overall MSI and prognosis but did find that a specific alteration (D16S402) was associated with a reduced DFS [260].

#### Prostate cancer

Prostate cancer is the most frequently diagnosed cancer in American men, with disease incidence increasing with age [261]. During the past 15 years, in the era of serum PSA screening, there has been a significant increase in the detection of prostate cancer [262-265]. Immunohistochemical staining and other methods designed to detect the cellular expression levels of PSA and prosaposin (PSAP) have not been generally successful in predicting outcomes in prostate cancer [266-272].

Both p21 and p27 proteins, members of the Cip/Kip family, have been studied as potential prognostic factors in prostate cancer [267]. Maintenance of p21 immunoreactivity is associated with prolonged DFS [273]. Loss of p27 expression has been associated with worsening disease outcomes in a number of studies [274-276]. Interestingly, although increased p16 has been associated with the presence of prostate cancer, this marker has not yet become a useful prognostic factor. For prostate cancer, the results of immunohistochemical analysis-based studies have been contradictory but have generally favored the result that overexpression of the HER-2/neu protein is associated with an adverse outcome [277-280].

Increased expression of bFGF has also been linked to adverse outcomes [281]. Overexpression of transforming growth factor β (TGFβ) has been implicated in the growth of prostate cancer cell lines and a significant reduction in DFS in clinical trials [282]. Up-regulation of vascular endothelial growth factor in prostate cancer has been associated with adverse outcomes in patients with clinically localized disease [283].

Multidrug resistance factors have been implicated in development of resistance of metastatic prostate cancer to conventional cytotoxic chemotherapy [284]. Several studies have linked overexpression of the antiapoptosis protein bcl-2 with decreased expression of the proapoptotic protein bax, and adverse outcomes in prostate cancer are associated with resistance to cytotoxic chemotherapy in patients with hormone-refractory disease [285,286].

The recently discovered proteasome inhibitor PS-341 (bortezomib (Velcade®)) has been associated with decreased production of bcl-2, inhibition of nuclear transcription factor κB (NFκB), and prevention of acquired resistance to chemotherapy in prostate cancer experimental systems [287]. NFκB overexpression in prostate cancer has recently been linked to adverse disease outcomes [288]. The NFκB complex has a role in cancer development and progression through its influence on apoptosis [289].

#### Glioma

Malignant gliomas consist of a broad range of histological entities, the majority of which respond poorly to standard therapeutic regimens. Although radiation and chemotherapy have been more successful to combat childhood medulloblastoma, with 5-year survival rates now as high as 70%–80%, the long-term side effects of these conventional treatments can be severe [290].

Cairncross et al. documented that oligodendrogliomas, with the loss of the short arm of chromosome 1 (1p), were preferentially chemosensitive when treated with a procarbazine, lomustine, and vincristine chemotherapy regimen [291]. These correlations with therapeutic sensitivity have been extended to other drugs such as temozolomide and procedures such as radiotherapy [292-294].

The expression level of MGMT and the methylation status of the MGMT gene promoter might have a predictive value in patients with glioblastoma treated with alkylating drugs such as temozolomide [93,295]. Hegi et al. studied the status of the MGMT gene promoter in tumors from patients enrolled in a large trial that investigated the role of concomitant temozolomide with radiation therapy vs radiation therapy alone [296]. The prognostic information conveyed by knowledge of the MGMT methylation status applied only to primary and not to recurrent tumors [297,298]. MGMT repairs O^6^-methylguanine DNA damage that is induced by alkylating agents such as temozolomide (currently the mainstay of anti-glioma chemotherapy), and strategies to overcome this resistance have been the focus of many studies [299,300].

Prognostically, amplification of EGFR could be associated with the age of the patient, and EGFRvIII expression might enable identification of a subgroup of more aggressive tumors [301,302]. EGFR-targeted drugs could be used to treat patients with glioblastomas that exhibit these characteristics in a similar fashion to some non-small-cell lung carcinomas that contain activating EGFR mutations and that show significant responses to the EGFR inhibitors erlotinib and gefitinib [303].

In a follow-up study, there were improved outcomes for patients with isocitrate dehydrogenase 1 (IDH1) and isocitrate dehydrogenase 2 (IDH2) tumor mutations, with a median OS of 31 months vs 15 months for patients with glioblastomas that lack these mutations, and 65 months vs 20 months for patients with anaplastic astrocytomas [304]. Recently, missense mutations in IDH1 were identified in a significant number of glioblastomas that tended to occur mostly in younger patients with more protracted clinical courses [305].

### Treatment modalities and adjustment

#### Efficacy and toxicity of current treatment

The best therapeutic options are drugs with high efficacy and minimal toxicity. An understanding of the cause of disease at the molecular level is needed to discover potential therapeutic targets eligible for drug intervention. Co-evolution of potential targets and drug development results in biomarker-driven therapies. Transformed cells become hypersensitive to specific inhibitors that target the deregulated oncogenic stimulus. This hypersensitivity is the basis of targeted therapies [306].

#### Pharmacogenetics to predict treatment outcome

Prediction of causative tumor abnormalities, markers of drug resistance, and pharmacokinetics markers are required to select and manage treatment to enhance efficacy of treatment and predict outcome. Pharmacogenetic (PGx) datasets provide information on variations of DME and drug targets, predicting efficacy, resistance, and/or toxicity of specific drugs in individuals. Databases of germline genetic variations associated with drug responses are available online and maintained through international initiatives. The PharmGKB Pharmacogenomics Knowledgbase (www.pharmgkb.org) is managed by the PharmGKB team at Stanford University, California, USA [307]. The Clinical Pharmacogenetics Implementation Consortium (CPIC) publishes peer-reviewed guidelines to translate this information to the clinic, including genotype-guided drug dosing. CPIC provides open-access, peer-reviewed, updated, evidence-based pharmacogenetic clinical practice guidelines [308]. In addition, the FDA provides drug labeling information that details the required or recommended genetic markers for informed prescribing.

The formulation of pharmacogenetic algorithms to assess the use of the correct drug at the correct dose for a particular individual is challenging due to complex genotype-phenotype associations, ethnic differences in allelic distribution, and variable penetrance of frameworks of variants. The FINDbase repository maintained by the GoldenHelix Institute of Biomedical Research [309] is an example of a large-scale initiative that aims to annotate allelic frequencies in different countries and to create a repository of ethnic differences. Provision of pharmacogenetic datasets is important, but the need of algorithms and guidelines is instrumental for clinical translation together with horizontal actions to provide education of health providers and proper tools to integrate the knowledge within the healthcare system.

Administration of drugs to a randomly selected cohort of cancer patients results in mild to lethal events due to varying toxicity. To reduce pharmaceutical toxicity, the implementation of genotype-guided dosing in healthcare systems is imperative. One such example is dihydropyrimidine dehydrogenase and the metabolism of 5-FU and pro-drugs. Fluoropyrimidines, including 5-FU, are widely used in the treatment of solid tumors and remain the backbone of many combination regimens. Despite their clinical benefit, fluoropyrimidines are associated with gastrointestinal and hematologic toxicities, which often lead to treatment discontinuation. 5-FU undergoes complex metabolism, and DPD is the rate-limiting enzyme of inactivation of 5-FU and its prodrugs. Several studies have demonstrated significant associations between severe toxicities by fluoropyrimidines and germline polymorphisms of DPD gene. To date, more than 30 SNPs and deletions have been identified within DPD, the majority of these variants have no functional consequences on enzymatic activity. However, the identification of deficient DPD genotypes might help identify poor-metabolizer patients at risk to develop potentially life-threatening toxicities after standard doses of fluoropyrimidines [2].

#### Mathematical modeling for oncology

Most processes found in medicine are non-linear, chaotic, and have a high level of complexity that impose difficulties to create a reliable mathematical model and in the use of information technology at all stages of the treatment process from the expression of the pathological processes to the implementation of therapeutic interventions. Creation of self-controlled systems based on forecasts of future medical errors is an important task.

To assess multi-parameter data, novel mathematical models are required to process the medical process as a complex system [310,311]. This process (cancer biomarkers panel longitudinal changes) is described by some of the primary indicators (imaging and immunohistochemical biomarkers). Thus, primary indicators and output rate are stochastic in nature and are presented as statistical information. The “best” mathematical model of the medical process is studied with a special algorithm to process statistical data.

Fractal geometry is a promising modality, especially the application of fractal analysis in complex systems of visual diagnostics including radiology and histology data, in order to expand its diagnostic capabilities by increasing the information content for intelligent decision modeling to reduce subjectivity in the perception and interpretation [312].

Processing of medical image analysis captures fractal parameters of these images to generate 3D vector and voxel models. This approach was successfully applied to hepatic ovarian and prostate tumors. Fractal geometry was estimated as 1.67 for hepatocellular carcinoma case, 1.72 for cholangiocarcinoma, 1.45–1.56 for complex cysts, and 1.15–1.35 for metastases [313].

### Current technologies: a brighter future

#### Single analysis for multi-cancer screening using metabolic information in blood

Many cancer-biomarkers and screening protocols have been proposed, but most of them are currently under clinical investigation. A lot of difficulties prevent them from becoming clinically useful, i.e., the heterogeneity of cell types, gene expression detected within each individual cancer patient, and different stages of disease progression. Therefore, the effectiveness of certain screening biomarkers or methods has to be varied with different types of cancer. For instance, colonoscopy, sigmoidoscopy, stool DNA, and fecal occult blood tests (FOBT) are currently recommended as CRC screening methods [314-316]. However, these methods involve either invasive, specialized procedures (e.g., colonoscopy) or low sensitivity (e.g., FOBT). Enormous efforts have been exerted to identify cancer biomarkers and to develop noninvasive methods for CRC screening. However, although serum CEA levels have been only accepted as a CRC tumor biomarker, they cannot be a screening marker for CRC [317]. Moreover, while advances in genomics, proteomics, and molecular pathology have suggested many candidate biomarkers with potential clinical value, trials to translate these research advances from bench to bedside have been disappointing. Currently, optimal blood markers for cancer screening are therefore lacking.

Profiling of the metabolic changes caused by cancer has become important for early detection. Indeed, metabolic profiling has shown potential for cancer screening [318-321]. To date, metabolic approaches that use liquid chromatography (LC)-mass spectrometry (MS) are very powerful to identify metabolites; however, studies demonstrated the limitation in the number of identified metabolites, usually no more than 100 distinct metabolites [322,323]. Such a low number of metabolites might not exactly reflect total metabolic changes in disease status. Furthermore, LC-MS is not acceptable as a high-throughput screening because of the relatively long analytical time for each sample.

We can postulate that valuable information that reflect metabolic changes that depend on the types of cancer might exist in the low-mass range less than 1,500 Thomsons (Th)—fibrinogen alpha chain, a very critical factor that represent inflammation, appeared at ~1,465 Th. Of course, one must always remember that a single m/z value cannot indicate a protein; only corresponding amino acid data unambiguously confirm the presence of a protein, peptide, etc. Most low-mass metabolic ions (LMIs) are mostly less than 800 Th. Systematic approaches to translate metabolic changes, particularly in blood, have already received attention; however, such information has not been systematically obtained with matrix-assisted laser desorption/ionization-time of flight (MALDI-TOF) MS, because a MALDI-TOF mass spectrum is neither as stable nor robust as other ionization methods. Furthermore, peak intensity does not accurately represent the quantity of a metabolite unless stable isotope-labeled internal standards are used for accurate quantification. However, MALDI-TOF has several advantages compared to electrospray-ionization (ESI)-based LC-MS with ion-trap MS, orbitrap MS, triple-TOF and quadrupole-TOF), which require les than a minute for one sample analysis, and more than 100 samples can be analyzed in a single target MALDI plate. Furthermore, even if the low-mass range contains numerous matrix peaks, they can be ruled out by their lower weighting factors in a computing process as with other metabolites affected by diet.

The first clinical trial of metabolic profiling (*LO*w-*M*ass ion discriminant *E*quation (LOME)) for CRC screening clearly showed its clinical potential [320]. LMI information from sera collected from healthy controls and from patients with CRC, gastric cancer (GC), breast cancer (BRC), non-Hodgkin lymphoma, ovarian cancer, and carcinoma *in situ* or advanced adenoma of the colon. A two-stage principle component analysis and novel algorithms were applied to LMIs that represented metabolic compounds in serum to develop a new concept of “LOME” for CRC, GC, and BRC screening. Finally, all sensitivities and specificities of LOMEs for CRC, GC, and BRC screening showed over 90% in validation clinical set. Such overall results strongly supported that LOME would be a powerful noninvasive tool with high sensitivity and specificity for cancer screening.

To construct the LOMEs, a high mass tolerance (up to 300 ppm) can be adopted. Therefore, the exact mass of selected LMIs for LOME construction could be confirmed and identified with high-accuracy MS. Furthermore, the pathological mechanisms responsible for altering the low-mass metabolites in CRC patients have to be clarified. Even with these disadvantages, clinical trials of LOME imply several important points superior to previous screening tools. First, combinations of multiple factors can better differentiate between cancer patients and healthy controls than one factor alone. The LOME method uses multiple LMIs in serum to achieve an excellent and robust cancer-screening power (for example, CRC LOMEs showed much higher sensitivity than FOBT). Second, each selected LMI can be identified and potentially useful as an individual biomarker for cancer screening. There is currently high interest in reliable and easy‐to‐measure cancer biomarkers. The three major LMIs in the discriminative biomarkers that composed CRC LOMEs were identified, e.g., fibrinogen alpha chain, which has previously been associated with various different cancer types. Third, LOME might have important implications to screen not only CRC but also other types of cancer. The versatility is the strongest advantage of LOME as well as the most-different aspect compared to other screening methods. New LOMEs could be constructed for a wide range of diseases to suggest a new paradigm for disease screening. Fourth, LOME can be constructed with the strict criteria for quality control of MALDI-TOF mass spectrum. Therefore, LOME can be the strong tool to extract useful information from inconstant MALDI-TOF mass spectrum.

Development of single analysis for multi-cancer screening with metabolic information in blood (e.g., LOME) will be required in the near future for better cancer treatment.

#### Dynamic analysis for monitoring therapy response using circulating cell-free tumor DNA

Circulating cell-free tumor DNA (ccftDNA) could be extracted and analyzed to investigate the dynamic evolution of tumor biology, particularly under the pressure of pharmacologic treatment. Indeed, significant amounts of ccftDNA are present in the plasma of cancer patients [324]. Because blood analysis of ccftDNA is relatively easy to perform with new technological and noninvasive platforms, ccftDNA represents a very attractive tool to detect the presence of mutations, particularly those associated with resistance to targeted agents, including KRAS [31]. ccftDNA dynamics can be easily followed and the appearance in the blood of a mutation associated with drug resistance is indicative of a genetic shift of the tumor and suggests the interruption of treatment and administration of alternative agents. Thus, less drug is given to patients that are becoming nonresponsive.

#### Imaging cancer for screening, diagnosis, staging and therapy response indicators

The ability to see the internal organs of the human body in a noninvasive way is a powerful diagnostic tool of modern medicine. Among these imaging modalities such as X-ray, magnetic resonance imaging (MRI), and ultrasound, MRI and ultrasound present much less risk of undesirable damage of both patient and examiner. Medical imaging permits the physician to plan the therapeutic procedure more accurately before carrying it out, to guide the intervention and more correctly locate the position of the interventional tool with respect to the anatomy, to monitor the intervention as it is being carried out, and to control the intervention for optimum results. Although static images are sufficient to plan radiation therapy or provide some anatomical information for surgery, real-time image guidance has been fundamental to the evolution of interventional procedures.

MRI is the most-effective technique to assess the type, degree of differentiation, presence or absence of lymphovascular invasion and lymph node involvement. Magnetic resonance spectroscopy (MRS) might support additional characteristics, namely ADC and total choline, that might be suggested in a role of predictive biomarkers. Diffusion-weighted MR imaging (DWI) is used as a response biomarker in patients who undergo chemoradiation for postoperative recurrences of cancer [325].

Positron emission tomography-computed tomography (PET/CT) has higher sensitivity and specificity than conventional anatomic modalities and is valuable to determine the extent of disease and detecting recurrent or residual tumor [326]. In locally advanced cervical cancer, ^18^ F-fluorodeoxyglucose (FDG) PET/CT has become important in the initial evaluation of disease extent. ^64^Cu-labeled diacetyl-di(N(4)-methylthiosemicarbazone) is taken up by hypoxic tissues, which might be valuable for prognostication and radiation treatment planning [327]. However, extensive use is subject to financial, legal, and radiation safety implications associated with whole-body PET/CT for cancer screening, diagnosing, staging, and restaging cancer and for monitoring treatment effects. In spite of advocating CT, PET, or PET/CT for whole-body screening, recommendations and decisions regarding cancer screening should be based on reliable data, not good intention, assumptions, or speculation [328]. For these reasons, actually, we still cannot consider PET as a perspective screening tool for a number of cancer locations.

Elastography data—promising noninvasive biomarker. Today, a new noninvasive method of examination, *sonoelastography* (SEG), which is based on the ultrasonic examination of tissue softness, is constantly under development. The technology is based on the fact of inverse scattering ultrasonic signal in mild compression and relaxed insonated tissue during the study [329]. Thomas et al. found that it was possible to diagnose malignant tumors of the cervix with elastography [330].

Angiogenesis visualization. Angiogenesis in cancer helps fulfill the metabolic demands of the progressing tumor and plays a critical role in tumor metastasis. Therefore, various imaging modalities have been used to characterize tumor angiogenesis. Micro-CT (μCT) is a powerful tool to analyze the tumor microvascular architecture at micron-scale resolution, MRI with its sub-millimeter resolution is useful for obtaining *in vivo* vascular data (e.g., tumor blood volume and vessel size index) [331]. However, integration of these microscopic and macroscopic angiogenesis data across spatial resolutions remains challenging. Laboratory biomarkers that represent vascularization might be conjoined with imaging data in particular for vasospasm and congestion assessment.

Optoacoustic imaging. The optoacoustic method was suggested in the diagnosis of cancer. The method is based on the ability of nanoparticles under the influence of near-infrared light to allocate heat. Nanoparticles can be passed around tissues and turned into sound waves that might be registered by an ultrasound receiver. Optoacoustic phenomena and other still hidden properties of nanomaterials open new views on personalized and predictive approach and could be the basis to create pharmacodynamic biomarkers ideal for use in diagnostics and prognostics and to suggest the likely outcome of a disease irrespective of treatment.

#### Development predictive biomarkers and relevant screening programs with focus on early diagnosis

Evidence-based public health has its roots in evidence-based medicine and arose as a need for evidence-based decision making in public health and radiology [332]. In consequence, trials that focus on intermediate endpoints (precancerous lesions or suitable biomarkers, e.g., ultrasound for atypical hyperplasia, prostate cancer, sonoelastography for breast, thyroid, soft tissue lesions) have become more widely accepted, especially because of a better understanding of the use of surrogate markers in cancer research and the fact that they can be carried out in a shorter period of time and with greater statistical power [333,334]. Collecting integrated biomarkers including imaging scans and data, obtained from relevant questionnaires to suggest relevant screening programs, allow outcomes such as avoid unnecessary imaging procedures. For instance, transvaginal ultrasonography has a poor positive predictive value but has a high negative predictive value to detect serious endometrial diseases in asymptomatic post-menopausal women [335,336].

#### Patient profile standardization

During the last decades, we were in a silent revolution based on the fact that all processes in image-based radiology have become entirely digital. Digitalization is the starting point for measurement. And just as measurement has allowed the natural sciences to progress, it enables medicine to progress. Relevant radiology (ultrasound, MRI, PET, CT, endoscopy) database modeling is performed with a scientific purpose to publish research results, and for processing of records, introspection, and the doctor of any other type of analysis (including financial). The key features of reliable statistical analysis are correct logging and archiving of research results.

#### Imaging modalities guidelines

A national effort to support comprehensive cancer control outlines national and state level success in comprehensive cancer control and provides a call to action to public, private, and non-profit organizations, governments of all levels, and individuals to renew their commitments to reduce the burden of cancer. The great majority of states have recommended European or national guidelines for use of imaging [337]. However, still the imaging modalities that involve ionizing radiation (radiography and nuclear medicine) were included in the great majority of guidelines (83%–92%), whereas non-ionizing radiology modalities (US, MRI) were present in only 75% of guidelines.

Hereby we recommend to develop guidelines to consider the PPPM approach with safe and effective screening and diagnostic algorithms and to consider needs of children and for pregnant women and elderly to adhere to the vision of many involved societies such as EPMA cancer and the specialized (such as neurology, urology, gynecology) societies in a multidisciplinary PPPM scope to suggest a balanced, well-reasoned, and evidence-supported application of different imaging modalities, by considering safe clinical validity, and to increase nonionized and effective modalities (such as ultrasound) in protocols. As further developments of cancer imaging might be considered for the improvement of cancer screening and diagnosis, we develop techniques upon utilizing physical properties like artifacts in diagnostic ultrasound and MRI to expand and improve image-guided treatments, robotics, and patient profile standardization, modeling the image-guided minimally invasive diagnostic and therapeutic interventional procedures with relevant education programs.

#### Nanotechnologies – the challenge for advanced diagnosis, treatment and prevention

Advances in nanoscience, nanotechnology, and nanomedicine lead to the construction of new materials and devices for various scientific and therapeutic purposes, which are applicable in molecular diagnostics, nanodiagnostics, and improvements in the discovery, design, and delivery of drugs, including nanopharmaceuticals. The application of nanoparticles that allow the combination of therapy and diagnosis, known as *theranostic*, has received increasing attention in biomedicine. Current evidence suggests that nanocrystalline ceria is a unique multifunctional material, which can be the basis to design a variety of drugs that promise for use in cancer therapy and diagnostics [338]. Many practical applications of ceria are based on its ability to protect living systems from oxidative stress of different origin, acting as an artificial analog of oxidoreductases, including superoxide dismutase, peroxidase, catalase, etc. The redox activity of nanocrystalline ceria is largely dependent on the particle size and composition of the environment, which makes it possible to fine-regulate the antioxidant and prooxidant properties of the preparations for redox therapy.

Nanocrystalline ceria are promising for application to tumors of viral origin. Of particular interest is the joint use of hybrid materials based on nanocrystalline ceria and protein anticancer drugs to increase the therapeutic effectiveness and reduce toxic side effects. The photoprotective and radioprotective effect of nanocrystalline ceria opens up broad prospects for its use in photodynamic therapy and radiotherapy of tumors. The pH-sensitive properties of nanoceria provide an opportunity to create a means of delivery of “smart” drugs, such as antitumor nanopreparations that release the drug in the right place at the right time [339].

Finally, nanocrystalline ceria, different CeO_2_-based solid solutions (including gadolinium (Gd)-containing solid solutions), and CeO_2_ nanoparticles with the surface modified with various ligands can be used for multimodal diagnosis of cancer, including malignant cell imaging and MRI and SPECT diagnostics. When choosing the material of nanoparticles for diagnosis, one should take into account, above all, the possibility to use them in existing diagnostic methods and equipment. For MRI, nanoparticles that contain Gd compounds [340] or superparamagnetic iron oxide Fe_3_O_4_ (SPIO) are used as a contrast agent. Nanoparticles of iodine derivatives, noble metals [341], some nontoxic heavy metal oxides, and rare earth compounds [342] might be considered for use as X-ray contrast agents.

For targeted delivery of nanoparticles to tumor, targeting ligands with tropism for tumor cells are used, including folic acid, VEGF, and sugars. Possibility of penetration of the drug into the cell is determined by the membrane affinity of nanoparticles; this affinity essentially depends on the surface charge of a nanoparticle. Nanoparticles of various types are widely used for drug delivery into tumor cells. The drug (e.g., an antibiotic, a cytotoxic agent or cytostaticcisplatin, and doxorubicin) can be located inside a nanoparticle and on its surface [343,344]. In the first case, such a “nanocapsule” should self-destruct in a tumor cell to release the drug [345]. In the second case, the medicine serves as yet another (third) surface ligand. A nanoparticle itself either is an inert carrier or also performs additional functions, for example, of contrast agent [343]. The drug on the surface of the nanoparticle can be additionally encapsulated, for example, into the cavity of the cyclodextrin molecule. In particular, cyclodextrin is used to fix the adamantane-PEG-transferrin conjugate on the surface of nanoparticles and to deliver small interfering RNAs (siRNAs) in gene therapy of cancer [339]. Among the clinically approved nanocomposites for drug delivery in chemotherapy of tumors are liposomal formulations of cytarabine, doxorubicin (Myocet®) and PEG-doxorubicin (Doxil®), daunorubicin (DaunoXome®), polymeric nanoparticles of methoxy_PEG-*L*,*D*-polylactide with taxol (Genexol_PM®) and PEG-*L*-asparaginase (Oncaspar®), paclitaxel albumin (Abraxane®) [345], among others.

#### Integration of omics to pilot the molecular networks for PPPM in human pituitary tumors: proof-of-principle models

Pituitary tumor is a complex whole-body disease that alters levels of gene (genome), mRNA (transcriptome), protein (proteome), and metabolite (metabolome) and that involves multi-factors, multi-processes, and multi-consequences [346]. Individual variation is involved in each stage of prediction/prevention, early-stage diagnosis/therapy, and late-stage diagnosis/therapy. The development of omics (genomics, transcriptomics, proteomics, and metabolomics) and systems biology has promoted one to change paradigms from a traditional single factor strategy to multi-parameter systematic strategy [346,347]. A new integrative opinion has been brought in the model of predictive screening and prognostic assessment of pituitary tumors that previously only depended on the changes of serum single-hormone change and pituitary imaging. The therapeutic model of cancer has changed from the general radiotherapy and chemotherapy to personalized strategy. The development of PPPM is substantially changing the understanding, prediction, prevention, and therapeutic model of pituitary tumors from a systematic to a comprehensive point of view.

From the point of view of systematic strategies for PPPM in pituitary tumors, it is necessary to systematically study the changes in genome, transcriptome, proteome, peptidome, and metabolome in individual pituitary tumor tissue and body-fluid (cerebral spinal fluid (CSF); serum/plasma) [347]. Systems biological strategies [348-353] will be used to integrate all experimental data and all clinical information of individual to propose the corresponding molecular networks specific to an individual pituitary tumor for efficient prediction screening, early-stage diagnosis, prognostic assessment, and individualized prevention and therapy.

The genome, transcriptome, proteome, peptidome, and metabolome are different among individual tumors and between tumor and normal, and the molecular network alters among individuals and between tumors and normal. Eight different levels of project studies will be necessary to achieve these objectives for PPPM practice in pituitary tumors.

#### Establish a reliable biobank of pituitary tumors

Two core aspects of the biobanking discipline are quality and good governance of biospecimen [354]. The reliable biobank and standardized procedure of collection and storage of biospecimen are the basis that guarantees one to realize large-scale molecular research in cancer [355-360]. For each pituitary tumor, its tumor tissue, CSF, blood, and all clinical information are required for eventual studies.

#### Establish molecular networks at the level of the genome of pituitary tumor

Breakthroughs of genomics technology have facilitated large-scale sequencing of the whole genome, genome-wide modification analysis, and transcriptome arrays in individual patients [361-363]. The complexity of those large-scale genome sequencing data might result from the heterogeneity of cell materials that might provide an averaging of results among tumor cells, stroma, endothelium and blood cells and lead to longstanding genomic instability [364,365]. High-throughput sequencing now allows one to sequence more numbers of patients and will facilitate the construction of genome-based molecular networks to allow better therapeutic decisions [361,366].

#### Establish molecular networks at the level of the transcriptome of pituitary tumor

Transcriptomics describe the global mRNA expression of a particular tissue and yield transcriptional difference data between two or more states [367,368]. It is essential to interpret the functional elements of the genome, reveal molecular constituents of cells and tissues, and understand the development of cancer. To construct transcriptome-based molecular networks, high-throughput platforms are today available to analyze and quantify the entire transcriptome profile of an organism [369].

#### Establish molecular networks at the level of the proteome of pituitary tumor

Proteomics is mainly used to identify, characterize, and quantify proteins in a defined biological system (organelle, cell, tissue, biofluid, or whole organisms) and has been considered as a powerful tool to study human tumors because of its ability to detect a large number of proteins in a short period of time [370-373]. The isobaric Target for Relative and Absolute Quantification (iTRAQ)-based quantitative proteomics is a commonly used quantitative proteomic methodology [374]. iTRAQ-based quantitative proteomics obtains protein expression profiles among individual tissue, blood cell, CSF, and plasma. Systems biology techniques are employed to construct proteome-based molecular networks.

#### Establish molecular networks at the level of the peptidome of pituitary tumor

Peptidomics is mainly used to identify, characterize, and quantify peptides in a body fluid and has been considered as a powerful tool to recognize a set of body fluid peptide biomarkers because body fluid contains peptides that result from tumor pathophysiological process, is a window that reflects tumor pathophysioligical states and its metabolic molecular networks, and would effectively serve for prediction, diagnosis, and prognosis assessment [375,376].

#### Establish metabolic networks at the level of the metabolome of pituitary tumor

Metabolomics is useful in the prediction of the effect of metabolic pathways on anticancer drugs in tumor patient [377]. It can measure global sets of low-molecular weight metabolites as indictors of physiological or pathological states. These large-scale metabolomic data can contribute to the construction of molecular interaction and gene regulatory networks able to predict drug effects [378,379].

#### Establish integrative molecular networks for pituitary tumor

Gene (genome), mRNA (transcriptome), protein (proteome), peptide (peptidome), and metabolite (metobolome) are mutually associated in an organism [346]. It is necessary to integrate different levels of molecule profile variations to construct integrative molecular networks for a pituitary tumor. Moreover, how those omics data-molecular networks link to clinical information is also important. Computation biology and systems biology can achieve those goals to systematically and comprehensively elucidate molecular mechanism and biomarkers for pituitary tumors.

#### Evaluate the molecular networks for prediction screening, early-stage diagnosis, prognostic assessment, and individualized prevention and therapy

The molecular networks are evaluated for accuracy, sensitivity, and specificity in the prediction screening, early-stage diagnosis, and prognostic assessment. The key molecular targets derived from molecular networks are useful for drug design and new drug development for efficient prevention and therapy.

A comprehensive and systematic analysis and validation of molecular networks at genome, transcriptome, proteome, peptidome, metabolome, and interactome levels completely clarify variations in molecular networks common and specific to the stratified and/or individualized pituitary tumors for the accurate and effective prediction, prevention, and personalized treatment for pituitary tumor to achieve the real PPPM practice. Moreover, a pituitary tumor is a model tumor in this section and all concepts, strategies, and techniques used for pituitary tumors can be easily translated to the study of other cancers.

#### Reliable cancer longitudinal in vivo animal models

Animal models have allowed the study of many diseases in the early stages, as well as investigation of the mechanisms of the pathogenesis and the effects of drug intervention. Mouse and rat models have been and still remain instrumental to reveal complexities of human cancer biology. An ideal animal model for any disease in humans should follow five characteristics: (1) mimic the human disease; (2) allow studies in chronic, stable disease; (3) produce symptoms that are predictable and controllable; (4) satisfy economical, technical, and animal welfare considerations; and (5) allow measurement of relevant cardiac, biochemical, and hemodynamic parameters. Because cardiovascular disease is uncommon in young humans but markedly increases with age, age-related changes of an animal should be considered.

The mouse (*mus musculus*) has emerged as the model organism of choice to study tumor development because the mouse and human are similar in many aspects such as basic physiology and genome size. The challenge for PPPM is to design the most clinically valid models inherent to the complexity of disease and similarity to human organism modeling, considering collateral cancer-associated diseases. Many studies with mouse and rat models are limited by the inability to gather experimental information *in vivo* and noninvasively. Small animal imaging solves many inaccuracies in the modeling [380].

Humanized mouse models of cancer, where human cells are introduced into immune-compromised mice and contribute/give rise to human tissues in the mouse, are of two types: (a) genetic-chimeras, where human genes/chromosomes are introduced into the mouse genome, and (b) cellular-chimeras. These developments could lead to better understanding of the various stages of cancer development and potentially lead to the development of more relevant pre-clinical models for therapeutic development [381].

### Implementing personalized medicine

The challenge of translating evidence-based discoveries to the clinical setting requires multidisciplinary approaches. The success of clinical studies depends on the proper classification of patients, use of the appropriate therapeutic groups for the studies, and proper design of the study. Meta-analysis between different studies is hindered by the lack of harmonization of variables. Drug efficacy and toxicity measures, the use of therapy modalities, and drug-administration protocols vary significantly. Hence a well-defined set of outcome measures should be adopted to allow meta-analysis [382]. Another variable is the clinical reporting of a phenotype. eTools should be adopted to harmonize the clinical reports, ideally with well-defined phenotypic and scoring methods. In addition, methods to correlate the studied marker with drug responses vary considerably. This can be easily overcome if the data is recorded using well-defined and set parameters that allow further analysis.

Education of healthcare providers is also an area that required continuous input. Implementation of genomic medicine suffers from the creation of a knowledge-base. Defining the required competencies within the healthcare system and creation of appropriate educational tools awaits a better integration of the data generated by genetic tests into the healthcare system. Currently, evidence-based associations are generated by comparing cohorts under genetic-guided therapy and those under clinical-guided care to measure the effectiveness of genetic tests. These comparisons are very informative, but delays treatment [383]. Due to this limitation, case–control studies are preferred. The translation of genetic data to the clinic needs harmonization of study designs [384] to allow data sharing and minimize the variability of pharmacogenetic results [385].

Implementation of specific tests should be justified on the basis of the clinical utility and the level of evidence. The CPIC, under the governance of the Pharmacogenomics Research Network (http://www.pgrn.org) and the Pharmacogenomics Knowledge Base [386], provides open-access, peer-reviewed, updated, evidence-based pharmacogenetic clinical practice guidelines [308]. Recently, the Italian Society of Pharmacology in conjunction with the Italian Association of Clinical Oncology issued a formal recommendation for the routine use of pharmacogenetic testing of DPD and UGT in clinical oncology practice [http://www.aiom.it].

The development of technologies and integrative information systems to provide a given healthcare system with optimized and sustainable testing protocols is required. The technology should provide results in a clear and conclusive report that is compatible with the healthcare data system. Adoption of electronic health records (EHRs) to store and interpret genetic results, based on clinically validated algorithms, provides a tool that will allow genetic-based informed decisions. The goal of this activity is to consolidate technology and methods and provide the healthcare system with a technology that will last for years and that will be fully supported for the same period of time. It is important to note that healthcare systems cannot change technologies frequently!

### Projected outcomes and recommendations

#### Background and general comments

The members of the EPMA PPPM in Cancer Group have years of experience in the many different fields of cancer that are discussed in this position paper. The group developed this report after EPMA/PPPM meetings and multiple rounds of e-mails to develop their individual and collective thoughts, outlines, and rough drafts.

#### Outlook

Cancer is a multifactorial disease that encompasses the genome, transcriptome, proteome, metabolome, and other to-be-discovered “-omes”.

Everybody would like to have a “magic bullet” biomarker that could be effectively used to quickly discover and accurately diagnose each cancer type and would lead to treatment that would effectively and completely obliterate each cancer at a very early stage. That desire to have a biomarker drives all of the clinical and basic research in cancer. However, as we delve more deeply into the complexity of cellular processes and develop more accurate and more precise analytical methods and pharmacological interventions, it becomes abundantly clear that, instead of only one biomarker, a panel of biological markers is needed to effectively diagnose and treat each cancer.

One must recognize the need for essential material to study. Cancer tissue is heterogeneous, and undergoes a quick evolution of mutation accumulation through genomic instability. Investigation of causative mutations in the development of the disease with tumor material is very complex. Understanding the etiology of primary lesions requires retrospective studies with matched blood and tissue at different stages of the tumor; hence, biobanks are important. Biobanking protocols should be harmonized and should include details of sample acquisition. To study proteins, it is important to rapidly place a surgical specimen into acid plus liquid nitrogen, to quickly stop metabolic processes, enzyme activity, and interconversions. Specialized protocols should be made available and properly implemented.

The future is quite bright and promising for biomarker discovery. Although it will not be easy to discover all needed biomarkers, clinical and research methods and instrumentation to study cancer become more powerful. Concomitantly, the development of effective pharmacology treatments will keep apace and derive input from the collection of -omics studies.

#### Recommendations

The recommendations of this PPPM in Cancer Group are to prepare a priority list of cancer risk factors that can be measured accurately using current technologies, formulate healthcare economic studies in different countries to define and justify expenses to measure risks as against healthcare costs, and prepare guidelines to monitor risks and define harmonized healthcare strategies to reduce the healthcare burden [387]. This potential deliverable ties in with various other actions that are required to ensure early diagnosis and a better quality of life for patients and to reduce cancer incidence and cancer mortality rate. Supportive actions include the development of guidelines to implement effective screening and diagnostic algorithms, expand and improve image-guided treatments, modeling the image-guided minimally invasive diagnostic and therapeutic interventional procedures, proper measures of efficacy and toxicity of current treatment including implementation of pharmacogenetic analysis, adjustment of treatment modalities based on pre-defined algorithms, and longitudinal actions such as the provision of relevant education programs, technological innovation, and medical research followed by evidence-based implementation to the clinic.

Multidisciplinary approaches are required as indicated in this position paper. We recommend a holistic approach to reduce cancer healthcare burden. For instance, other disorders and their treatment are also a risk factor for cancer initiation. Chronic inflammation of the bowel is one example used in this paper. The use of bottom-up approach in treatment adjustment is considered as safe for the patient, but evidence should be collated to understand if this approach promotes development of cancer. Hence, we recommend to develop guidelines to stratify patients using well-defined biomarkers, measuring risks associated with cancer development, and provide actionable solutions to reduce such risks. Implementation of personalized medicine is challenging, and translating evidence-based discoveries to the clinic requires well-defined guidelines.

Many cancers will greatly benefit from a concerted effort to significantly and quickly increase research funding in cancer, develop new panels of biomarkers, develop more-effective pharmacological agents, and increase each individual’s participation in their healthcare [388]. The PPPM concept is gaining worldwide acceptance and momentum, and results are impressive. Because of the extensive worldwide momentum of clinical and basic research in cancer, the time is ripe for a quantum increase in the amount of money devoted to investigator-initiated cancer research. That recommendation is obviously not only scientific or clinical but also political because it clearly invites everybody involved with cancer to participate in that exciting activity. Virtually, every person knows somebody who has, or has died from, a cancer. Most people feel helpless, confused, angry, etc. when confronted with a cancer in their life or in somebody close to them. The most effective approach to increase funding is for each person, patient, family member, researcher, neighbor, grandparent, interested person, etc. to write directly to representatives in their government’s funding agency. In the U.S.A., the most-effective person to write to is a representative and senator. We know that this method works because the doubling of the U.S.A. National Institutes of Health research budget 15 years ago was due in large part to the widespread outpouring of letters written to the congress by researchers and those touched by cancer.

## Conclusion

The overall goal of EPMA and PPPM cancer is to significantly decrease the number of deaths, continuously improve early detection, develop state-of-the-art diagnosis and treatment, improve the quality of life of patients, and develop easy-to-use kits for diagnosis as clearly stated in the General Report and Recommendations in Predictive, Preventive and Personalised Medicine 2012: White Paper of the European Association for Predictive, Preventive and Personalised Medicine [389]. It is important to study the mechanisms that lead to cancer in order to effectively prevent it. Much more basic research is required. Each individual is, by definition, unique. Each person’s cancer is unique. Each treatment is unique. It is important to predict therapy resistance and relapse. Each individual will respond differently to treatment. There is no “one treatment fits all cases.” Each patient must be monitored to accurately determine their response to treatment.

In summary, while discussing implementation of personalized medicine, governments and healthcare systems should be mindful of the potential for flaws in delivery of adequate care and thus ensure that new developments in therapy are available to all patients and not just to a select few. If this issue is addressed comprehensively by EPMA in developed nations (e.g., the EU) to start with, we can then be confident of rolling this approach out to less wealthy nations, globally.
